# *Streptococcus pneumoniae* potently induces cell death in mesothelial cells

**DOI:** 10.1371/journal.pone.0201530

**Published:** 2018-07-30

**Authors:** Rabab Rashwan, Julius F. Varano della Vergiliana, Sally M. Lansley, Hui Min Cheah, Natalia Popowicz, James C. Paton, Grant W. Waterer, Tiffany Townsend, Ian Kay, Jeremy S. Brown, Y. C. Gary Lee

**Affiliations:** 1 Centre for Respiratory Health, University of Western Australia, Perth, Western Australia, Australia; 2 Department of Microbiology and Immunology, Faculty of Medicine, Minia University, Minya, Egypt; 3 School of Medicine & Pharmacology, University of Western Australia, Perth, Western Australia, Australia; 4 Department of Pharmacy, Sir Charles Gairdner Hospital, Perth, Western Australia, Australia; 5 Research Centre for Infectious Diseases, Department of Molecular and Cellular Biology, University of Adelaide, Adelaide, South Australia, Australia; 6 Respiratory Department, Royal Perth Hospital, Perth, Western Australia, Australia; 7 Dept of Microbiology & Infectious Diseases, Royal Perth Hospital, Perth, Western Australia, Australia; 8 Centre for Inflammation and Tissue Repair, UCL Respiratory, University College London, London, United Kingdom; 9 Dept of Respiratory Medicine, Sir Charles Gairdner Hospital, Perth, Western Australia, Australia; Instituto Butantan, BRAZIL

## Abstract

Pleural infection/empyema is common and its incidence continues to rise. *Streptococcus pneumoniae* is the commonest bacterial cause of empyema in children and among the commonest in adults. The mesothelium represents the first line of defense against invading microorganisms, but mesothelial cell responses to common empyema pathogens, including *S*. *pneumoniae*, have seldom been studied. We assessed mesothelial cell viability *in vitro* following exposure to common empyema pathogens. Clinical isolates of *S*. *pneumoniae* from 25 patients with invasive pneumococcal disease and three reference strains were tested. All potently induced death of cultured mesothelial cells (MeT-5A) in a dose- and time-dependent manner (>90% at 10^7^ CFU/mL after 24 hours). No significant mesothelial cell killing was observed when cells were co-cultured with *Staphylococcus aureus*, *Streptococcus sanguinis* and *Streptococcus milleri* group bacteria. *S*. *pneumoniae* induced mesothelial cell death via secretory product(s) as cytotoxicity could be: i) reproduced using conditioned media derived from *S*. *pneumoniae* and ii) in transwell studies when the bacteria and mesothelial cells were separated. No excess cell death was seen when heat-killed *S*. *pneumoniae* were used. Pneumolysin, a cytolytic *S*. *pneumoniae* toxin, induced cell death in a time- and dose-dependent manner. *S*. *pneumoniae* lacking the pneumolysin gene (D39 ΔPLY strain) failed to kill mesothelial cells compared to wild type (D39) controls, confirming the necessity of pneumolysin in D39-induced mesothelial cell death. However, pneumolysin gene mutation in other *S*. *pneumoniae* strains (TIGR4, ST3 and ST23F) only partly abolished their cytotoxic effects, suggesting different strains may induce cell death via different mechanisms.

## Introduction

Bacterial pleural infection is a centuries-old disease and the global incidence continues to rise [1]. Community-acquired pneumonia affects over 5 million people each year in the United States [2, 3]. Of those, 20–40% will be complicated by development of a parapneumonic effusion [4], which can be secondarily infected by bacteria (pleural infection) and may present with frank pleural pus (empyema). Pleural infection is associated with a high (~20%) mortality in adults [5].

*Streptococcus pneumoniae* is the commonest cause of empyema in pediatric populations [6, 7] and the second most common in adults [1]. The *Streptococcus milleri* group (*S*. *anginosus*, *S*. *intermedius* and *S*. *constellatus*) is another leading cause of adult empyemas [8–10] whereas *Staphylococcus aureus* is the most frequent cause of hospital-acquired empyemas [11, 12].

Mesothelial cells line the pleural cavity and are the predominant cell type in the pleura. During infection, the mesothelium represents the first line of defense by acting as a surface barrier to invading pathogens [13]. Our previous animal model data showed that, following aspiration into the lung, *S*. *pneumoniae* infects the lung parenchyma and spreads rapidly toward the lung surface where it can disrupt the mesothelial barrier and invade the pleura to produce an empyema [14].

Despite the prevalence and importance of pleural infection, few other studies have investigated the effect of common bacterial pathogens (especially *S*. *pneumoniae*) on pleural mesothelial cells. In this study we aimed to characterize the effect of bacterial exposure (especially *S*. *pneumoniae*) on pleural mesothelial cell survival *in vitro*. The role for the secreted cytolytic toxin pneumolysin during mesothelial cell death was also assessed using genetically modified strains and recombinant pneumolysin.

## Material and methods

### Mesothelial cells

The SV40-transformed human mesothelial MeT-5A cell line was obtained from the American Tissue Culture Collection (Manassas, VA, USA; #CRL-9994). Cells were maintained in Dulbecco’s Modified Eagle Medium (DMEM) supplemented with 4 mM L-glutamine, 0.2 μg/ml streptomycin, 0.2 μg/ml penicillin and 10% (v/v) fetal calf serum (FCS).

### Bacterial strains and culture

Bacterial reference strains were obtained from ATCC (Table 1). *S*. *pneumoniae* clinical isolates were cultured from patients with invasive disease and included 22 blood and 3 pleural fluid isolates (Table 2). All clinical isolates were collected from Royal Perth Hospital (Perth, Western Australia), except for WCH43, which was provided by Professor James Paton (University of Adelaide, South Australia). Wild type *S*. *pneumoniae* D39, TIGR4, ST3 and ST23F strains and their pneumolysin-negative derivatives (referred to as ΔPLY) were kindly provided by Professor Jeremy Brown (University College London, London, UK) [15, 16]. Ethics approval was obtained from the University of Western Australia Institutional Biosafety Committee (Approval number RA/5/1/445).

**Table 1:** List of *S*. *pneumoniae* reference strains used in this study.

Strain designation	Description	Source
***Streptococcus pneumoniae***		
D39	Capsular serotype 2	[15, 16]
WCH43	Capsular serotype 4	Women’s and Children’s Hospital, North Adelaide, Australia
TIGR4	Capsular serotype 4	ATCC® BAA-334™
CIP 104225	Capsular serotype 3	ATCC® 6303™
***Staphylococcus aureus***		
Seattle 1945	Clinical isolate obtained in Seattle in 1945	ATCC® 25923™
Smith	Clinical isolate	ATCC® 13709™
***Streptococcus anginosus***		
NCTC 10713	Isolated from human throat tissue	ATCC® 33397™
***Streptococcus intermedius***		
VPI 3372A	Type strain	ATCC® 27335™
***Streptococcus constellatus***		
VPI 3810	Isolated from a patient with purulent pleurisy	ATCC® 27823™
***Streptococcus sanguinis***		
DSS-10	Isolated from a patient with sub-acute bacterial endocarditis	ATCC® 10556™

**Table 2:** List of *Streptococcus pneumoniae* clinical isolates used in this study.

Strain designation	Capsular serotype	Source
RPH 31856	1	Pleural fluid
P11-9566884P	1	Blood culture
P12-3039379M	8	Blood culture
P12-3035869G	8	Blood culture
P11-9618385UM	8	Blood culture
P12-9552330W	6B	Blood culture
P11-9608890J	6C	Blood culture
P11-9624937G	10A	Blood culture
RPH 31337	11A	Pleural fluid
P11-9653902K	11A	Blood culture
P11-9613539M	12F	Blood culture
P11-9594082Q	16F	Blood culture
RPH 31450	19A	Pleural fluid
P11-9645796U	19A	Blood culture
P11-3086723K	19A	Blood culture
P11-9607769H	19A	Blood culture
P11-9591891X	19A	Blood culture
P11-3048281G	19A	Blood culture
P11-9585892W	19A	Blood culture
P11-3047887E	19A	Blood culture
RPH 31018	19F	Blood culture
P12-3029494R	21	Blood culture
P12-3028290U	22F	Blood culture
P11-9611457G	22F	Blood culture
P12-9562415S	35B	Blood culture

Streptococcus species were cultured in Todd Hewitt broth containing 0.5% yeast extract (THY), while *S*. *aureus* strains were grown in Luria Bertani medium. Bacteria were stored in broth containing 20% (v/v) glycerol at -80°C and directly sub-cultured onto blood agar plates for 18–24 hr at 37°C in 5% (v/v) CO_2_ before use. For the ΔPLY strains, sub-culturing was performed using blood agar plates supplemented with 0.2 μg/mL erythromycin. For experimentation, bacterial suspensions were prepared in 0.85% (w/v) saline to a turbidity of 0.5 McFarland using a Sensititre Nephelometer (Thermo Scientific; Waltham, MA, USA). Bacteria were also subject to heat-killing at 95°C for 1 hr. Successful heat-killing and viability of the live bacteria was verified by plate counts. Briefly, ten-fold dilutions of each bacteria ranging from 10–1 to 10–6 colony forming units (CFU)/mL were prepared in saline, with 20 μL spotted onto blood agar plates, and incubated overnight at 37°C. The following day, the number of CFU per 20 μL was counted and the CFU/mL calculated.

### Preparation of *S*. *pneumoniae* conditioned media

*S*. *pneumoniae* was directly sub-cultured from blood agar plates into DMEM and incubated overnight in a shaking incubator at 200 rpm at 37°C. The conditioned media was filter-sterilized using a 0.2 μm pore size filter. For each experiment, the sterility of the conditioned media was confirmed by plating onto blood agar.

### Recombinant native pneumolysin

Recombinant native pneumolysin was purified and assessed for hemolytic activity as previously described [17]. The preparation contained an activity of 380,000 hemolytic units per mg protein.

### Bacterial infection of mesothelial cells

For all experiments, MeT-5A cells were grown to confluence in 24-well plates and deprived of serum and antibiotics 24 hr prior to stimulation. Cells were treated with live or heat-killed bacteria (at 10^5^, 10^6^ and 10^7^ CFU/ml in 500 μL), conditioned media or recombinant pneumolysin for up to 24 hr. Transwell experiments were performed where MeT-5A cells were cultured in 24-well plates and 200 μL of a *S*. *pneumoniae* suspension (2 x 10^7^ CFU/mL) was transferred into the upper chamber of Corning® Transwell® polyester membrane cell culture inserts (6.5 mm diameter, 0.4 μm pore size; Sigma-Aldrich). The transwell design did not allow physical contact between the mesothelial cells and bacteria. Viability of the bacteria in the upper chamber and their exclusion within the lower chamber was verified by plating onto blood agar using aliquots of the supernatant from each chamber. At the conclusion of each experiment the cells were analyzed for viability using flow cytometry.

### Determination of cell viability

Cell viability was assessed using a LIVE/DEAD^®^ Fixable Dead Cell Stain kit (Invitrogen; Victoria, Australia) according to the manufacturer’s instructions. Briefly, cells were harvested by trypsinization and washed with PBS. Cells were then stained with 1:1000 diluted green fluorescent reactive dye for 30 min, washed with PBS and fixed in 4% (w/v) paraformaldehyde for 15 min. Following this, cells were resuspended in PBS and fluorescence detected by flow cytometry using a 530/30 bandpass filter. Data (each 10,000 events) were acquired and analyzed using a BD FACSCalibur with FACSDiva version 5 software (BD Bioscience; San Jose, CA, USA).

### Hemolysis assay

Cytolytic activity of the clinical isolates, reference, wild type and ΔPLY *S*. *pneumoniae* strains was determined semi-quantitatively using an erythrocyte hemolysis assay [18]. Bacterial suspensions were made in PBS and pelleted at 10,000 x *g* for 5 min. The pellet was lysed using 1% (w/v) sodium deoxycholate and stored at -20°C until required. Protein concentrations of the clarified lysates were determined using the DC Protein Assay kit (Bio-Rad: Hercules, CA, USA), according to the manufacturer’s instructions. A 5% (v/v) erythrocyte suspension was prepared from whole human blood and activated for 15 min at room temperature following the addition of 20 mM 2-mercaptoethanol. A 100 μL aliquot of each lysate (50 μg/mL) was serially diluted in PBS and an equal volume of erythrocyte suspension was added. The samples were incubated at 37°C for 30 min and centrifuged at 800 x *g* for 5 min. The absorbance of the supernatant at 540 nm was then measured. Erythrocytes treated with PBS alone were included as a measure of background absorbance and used to correct absorbance values of samples treated with lysates.

### Western blot analysis

Bacterial cell lysis was performed using RIPA buffer containing 1X protease and phosphatase inhibitor cocktail (Thermo Fisher). Lysates were kept on ice for 30 mins with vortexing every 10 mins then centrifuged at 4°C 14,000 x *g* for 20 minutes before supernatants were transferred to a clean microfuge tube and stored at -20°C. Protein was quantified using Coomassie Plus reagent as per manufacturer’s instructions (BioRad). Ten micrograms of protein was denatured by heating at 95°C for 5 minutes, separated on 4–12% Bis-Tris gels (Invitrogen) and transferred onto nitrocellulose membranes using the iBlot system (Invitrogen). Membranes were blocked for 1 hour at room temperature in 5% skim milk/TBS-T (TBS with 0.05% Tween 20). Membranes were then incubated with anti-pneumolysin (sc-80500; 1:500: Santa Cruz Biotechnology) in TBST containing 5% skim milk powder on a roller overnight at 4°C. After washing a further 3 times with TBST for 5 minutes, blots were incubated with rabbit anti-mouse HRP-conjugated secondary antibody (ab6728: 1:10,000 Abcam) for 1 hour at room temperature. Membranes were visualized using the Pierce™ ECL Western Blotting Substrate (Pierce) and hyperfilm ECL (GE Healthcare, UK).

### Statistical analysis

Data are presented as the mean ± standard error of the mean (SEM). Student’s *t* test with Bonferroni correction was used to compare differences between two treatment groups. A *p* value <0.05 was considered statistically significant. Analyses were conducted using GraphPad Prism 4.0 (La Jolla, CA, USA).

## Results

### *S*. *pneumoniae* potently induces death of pleural mesothelial cells

**Fig 1:**
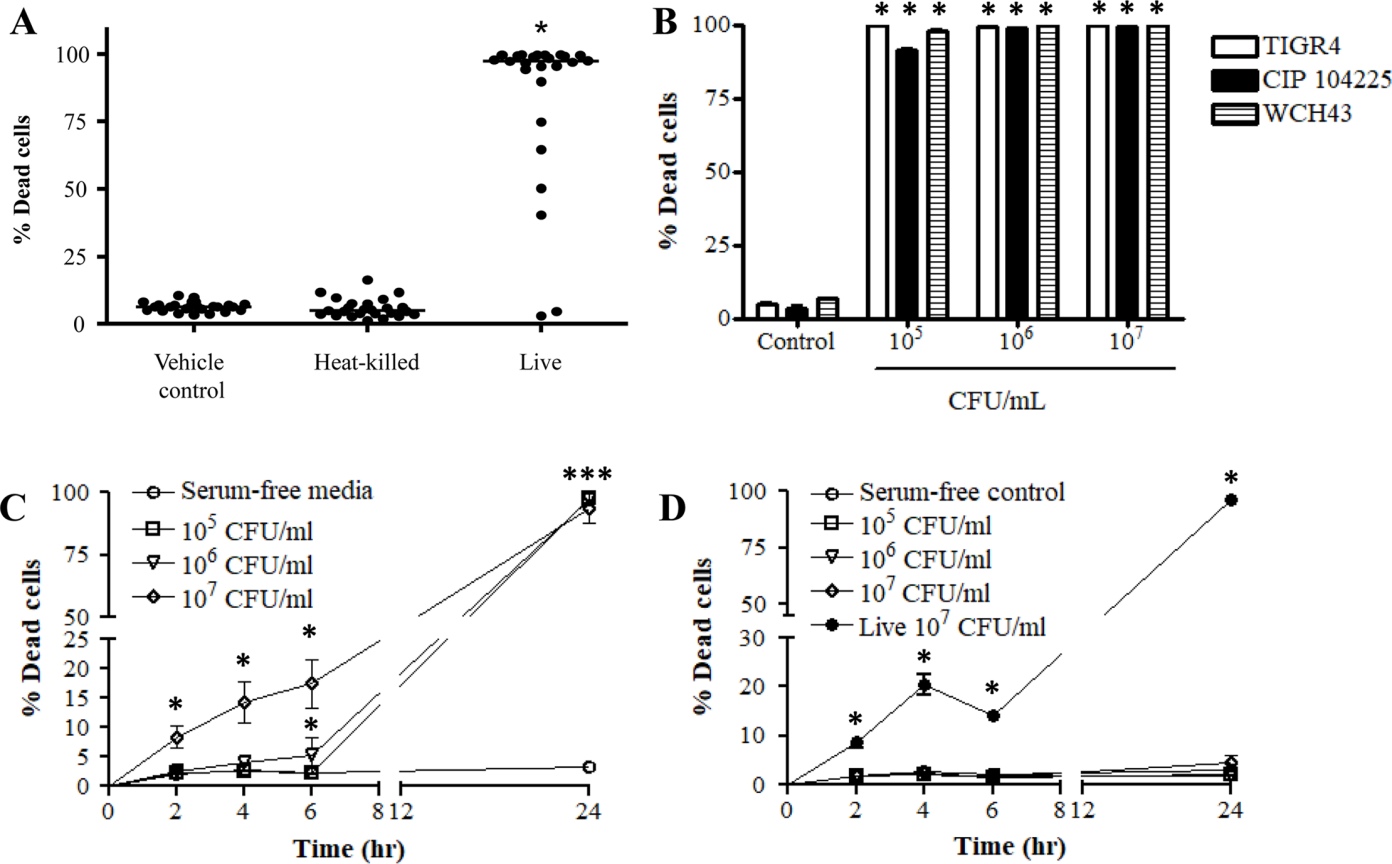
Infection with *Streptococcus pneumoniae* induces death of pleural mesothelial cells. A) The effect of *S*. *pneumoniae* on mesothelial cell death was assessed using 25 clinical isolates obtained from patients with invasive pneumococcal disease. B) MeT-5A cells were treated with increasing doses of live *S*. *pneumoniae* reference strains. C) Time course of MeT-5A cell death following *S*. *pneumoniae* TIGR4 infection was also assessed. D) Time course of MeT-5A cell death following co-culture with *S*. *pneumoniae* TIGR4 following heat-treatment at 95°C for 1 hr and viability determined at various time points up to 24 hr post-infection. * Denotes significantly higher than vehicle control and heat-killed bacteria.

To investigate the effect of bacteria on pleural mesothelial cell survival, confluent MeT-5A monolayers were co-cultured with a range of common bacterial pleural empyema pathogens. Twenty five clinical isolates of *S*. *pneumoniae* isolated from patients with invasive pneumococcal diseases were tested (Table 2), of which 23 induced significant killing of pleural mesothelial cells (median % dead cells at 10^7^ CFU/mL, 97%; p < 0.0001 compared to vehicle controls). Further, when heat-killed, none of the 25 clinical isolates tested were able to induce mesothelial cell death (Fig 1A).

In subsequent experiments 3 reference strains were used (Table 1) to explore the mechanisms of *S*. *pneumoniae*-induced cell death. Following infection for 24 hr, *S*. *pneumoniae* TIGR4, CIP 104225 and WCH43 strains potently induced death of MeT-5A cells (Fig 1B). Mesothelial cell death was observed at all bacterial doses used and no significant difference in cell death was seen between doses higher than 10^5^ CFU/mL. At 10^7^ CFU/mL, TIGR4, CIP 104225 and WCH43 strains killed 93.7%, 99.2% and 99.9% of MeT-5A cells, respectively (p < 0.0001 compared to vehicle control) (Fig 1B).

In subsequent experiments, a time course response to infection with the *S*. *pneumoniae* TIGR4 strain was also determined. As shown in Fig 1C, a significant increase in cell death was seen as early as 2 hr post-infection in cells treated with the highest bacterial dose (10^7^ CFU/mL). This response continued to increase for all subsequent time points measured (2, 4, 6 and 24 hr post-infection: 8.2%, 14.3%, 17.4% and 93% of total; p < 0.05 for all time points). In contrast, cell death was only significantly induced at the 24 hr time point in cells treated with 10^5^ or 10^6^ CFU/mL (p < 0.0001; Fig 1C).

### Heat-killing of *S*. *pneumoniae* abolished its ability to induced mesothelial cell death

To determine whether *S*. *pneumoniae*-induced cell death was dependent on the presence of live bacteria, *S*. *pneumoniae* (TIGR4 strain) was heat treated prior to co-culture with mesothelial cells. Compared to live bacteria, heat-killed TIGR4 failed to induce MeT-5A cell death at all concentrations used and at all time points measured (% dead cells 24 hr post-infection at 10^7^ CFU/mL; live, 94%; heat-killed, 4.5%; p < 0.0001) (Fig 1D).

### Infection with other empyema-causing bacterial strains did not induce mesothelial cell death

**Fig 2:**
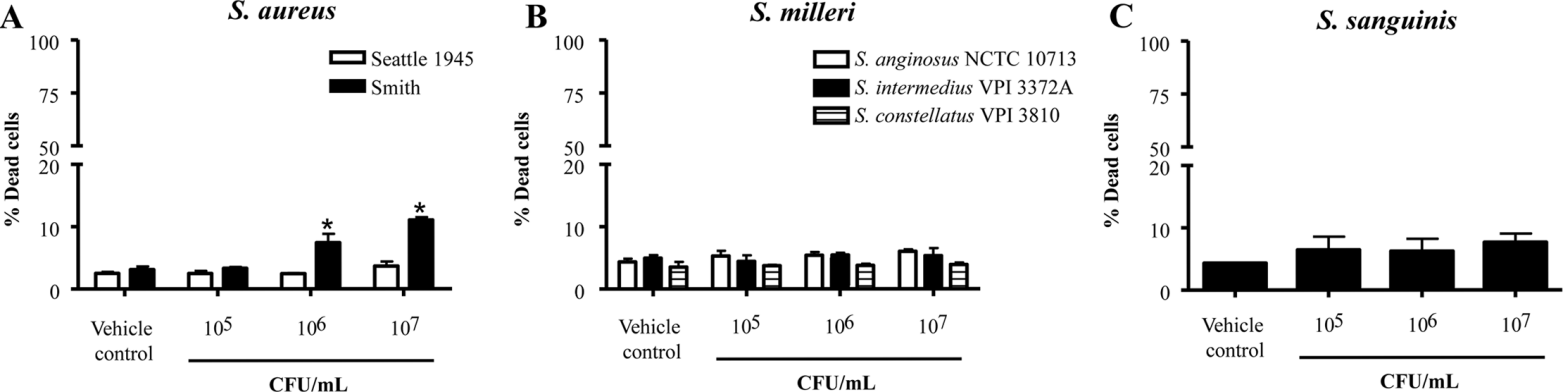
Infection of mesothelial cells with non-*S*. *pneumoniae* bacterial strains does not potently induce cell death. A-C) MeT-5A cells were treated with increasing doses of live *S*. *aureus* (A), *S*. *milleri* group bacteria (B) or *S*. *sanguinis* (C) and cell viability was assessed. * Denotes significantly higher than vehicle control.

Treatment of MeT-5A cells with *S*. *aureus* Smith strain induced a very mild dose-dependent increase in cell death up to 11% at 10^7^ CFU/mL (p < 0.001; Fig 2A). In contrast, *S*. *aureus* Seattle 1945 strain, S. *milleri* bacteria and *S*. *sanguinis* had negligible effect on mesothelial cell viability at all concentrations tested (Fig 2A–2C).

### *S*. *pneumoniae*-induced mesothelial cell death is mediated via a secreted bacterial product

**Fig 3:**
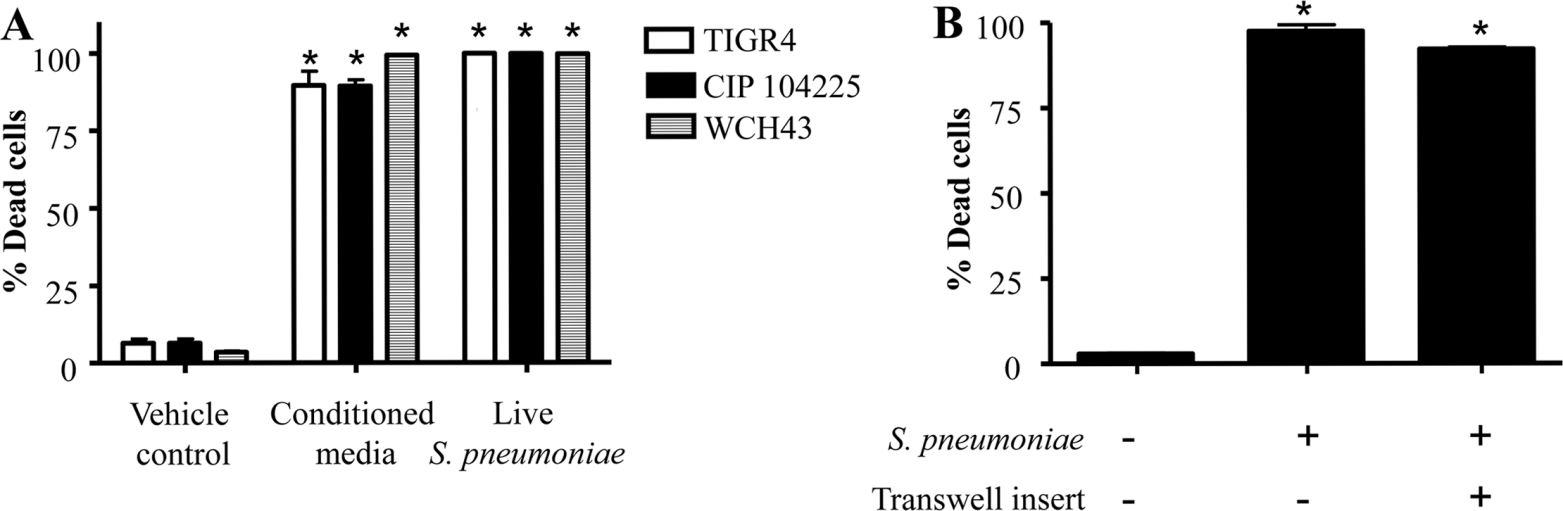
*S*. *pneumoniae*-induced mesothelial cell death is mediated by a bacterial secreted product. A) MeT-5A cells were incubated with cell-free bacteria conditioned media. B) *S*. *pneumoniae* was loaded into the top chamber of a Transwell® insert and viability of MeT-5A cells was determined in the bottom chamber. Cell viability was assessed 24 hr post-treatment. * Denotes significantly higher than vehicle control.

In subsequent experiments we assessed the potential role for a secreted product in *S*. *pneumoniae*-induced mesothelial cell death. Cell-free conditioned media derived from *S*. *pneumoniae* TIGR4, CIP 104225 and WCH43 strains significantly induced death of MeT-5A cells compared to vehicle controls (Fig 3A; p < 0.0001). This effect was similar in potency to treatment with live *S*. *pneumoniae*. To further explore this observation, MeT-5A cells were co-cultured with the *S*. *pneumoniae* TIGR4 strain using Transwell® inserts. As shown in Fig 3B, culture of TIGR4 in the top chamber of a Transwell® insert still resulted in death of the mesothelial cell layer in the bottom chamber (p < 0.0001). Media obtained from the bottom well was devoid of bacteria.

### *S*. *pneumoniae-*induced mesothelial cell death is partly mediated by pneumolysin

**Fig 4:**
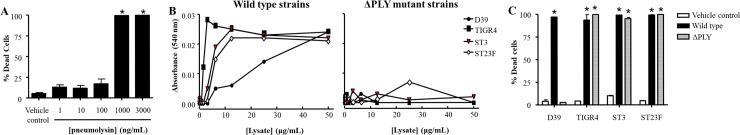
The role of pneumolysin in *S*. *pneumoniae*-induced cell death. A) MeT-5A cells were treated with a purified, recombinant preparation of pneumolysin and viability determined 24 hr post-treatment. B) Lysates prepared from wild type *S*. *pneumoniae* strains and their pneumolysin-negative derivatives (ΔPLY) were assessed for cytolytic activity using an erythrocyte hemolysis assay. C) MeT-5A cells were infected with wild type and ΔPLY *S*. *pneumoniae* strains and viability determined 24 hr post-infection. * Denotes significantly higher than vehicle control.

Pneumolysin is a potent cytolytic toxin produced by all clinically isolated *S*. *pneumoniae* strains and represents a major virulence determinant in pneumococcal disease [19]. Given this, the role of pneumolysin in *S*. *pneumoniae-*induced mesothelial cell death was investigated. First, cultured mesothelial cells were treated with recombinant pneumolysin to assess for toxin-induced lysis. As shown in Fig 4A, pneumolysin potently induced MeT-5A cell death at 1000 and 3000 ng/mL (p < 0.0001 compared to the vehicle control).

We then employed a variety of wild type and pneumolysin-negative mutant strains to verify the importance of the toxin in this process. All parental wild type strains produced cytolytic toxins, as assessed by an erythrocyte hemolysis assay. In contrast, none of the ΔPLY strains were cytolytic, (Fig 4B). Wild type *S*. *pneumoniae* D39 potently killed the mesothelial cells, but the D39 ΔPLY failed to induce mesothelial cell death (Fig 4C), confirming that pneumolysin mediates the D39-induced cell killing. In contrast, TIGR4, ST3 and ST23F and their respective ΔPLY strains were equally potent in killing mesothelial cells, suggesting these strains employed a different mechanism (other than pneumolysin) in inducing cell death.

**Fig 5:**
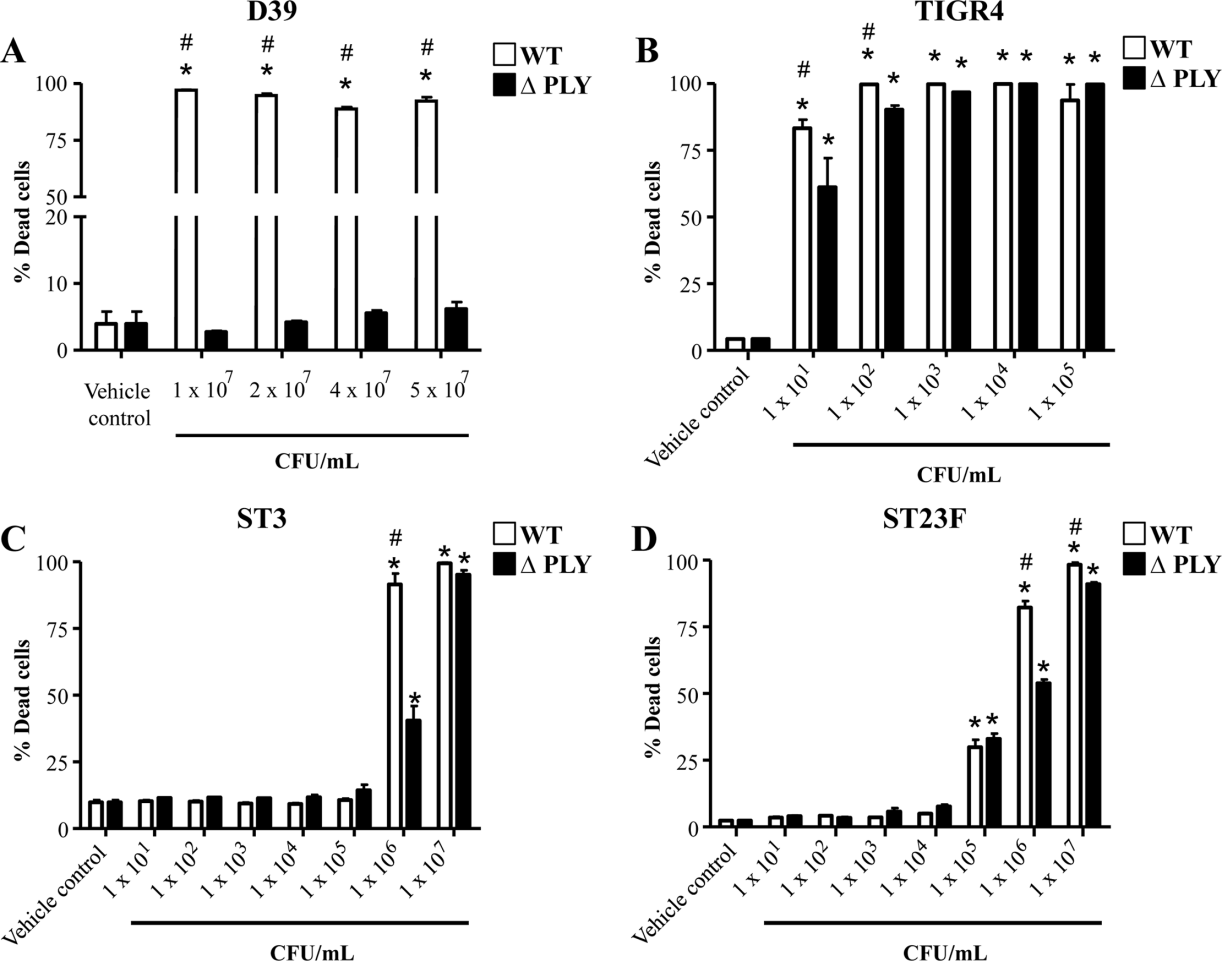
Dose response of wild type and pneumolysin-negative mutant *S*. *pneumoniae* strains on pleural mesothelial cell death. A-D) MeT-5A cells were treated with various concentrations of D39 (A), TIGR4 (B), ST3 (C) or ST23F (D) wild type and pneumolysin mutant (ΔPLY) strains and viability determined 24 hr post-infection. * Denotes significantly higher than vehicle control. # Denotes significant higher than ΔPLY strain.

**Fig 6:**
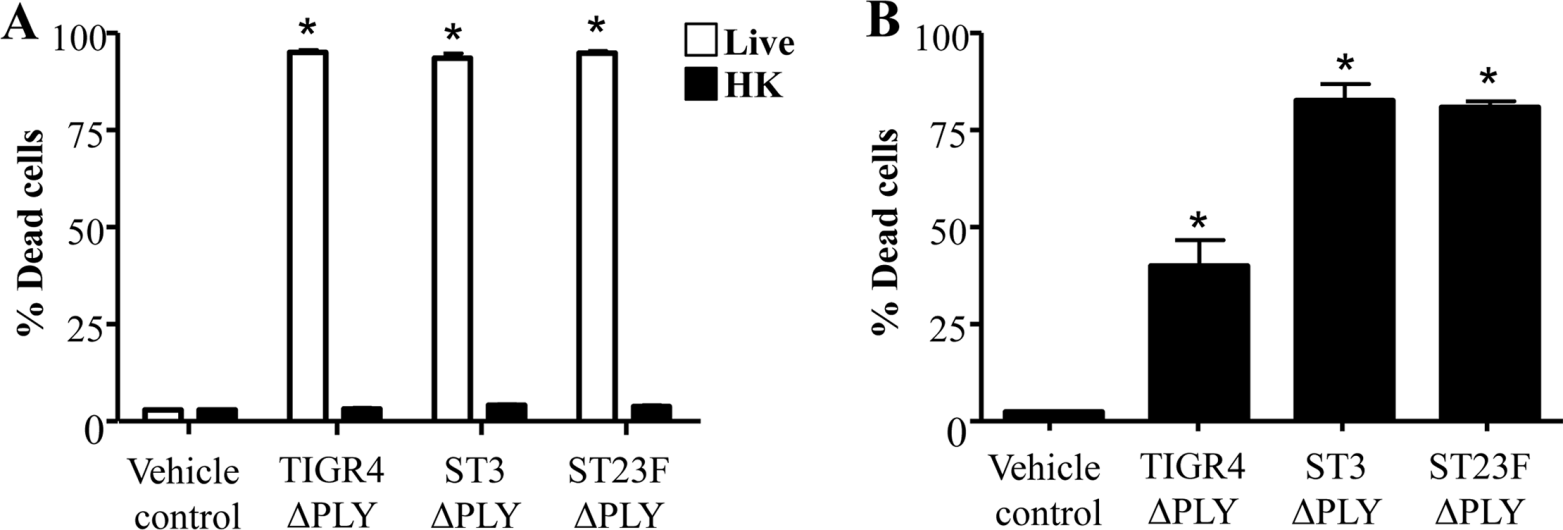
The effect of heat-killing and conditioned media derived from pneumolysin mutant *S*. *pneumoniae* strains on pleural mesothelial cell death. A) MeT-5A cells were incubated with either live (open bars) or heat-treated (closed bars) *S*. *pneumoniae* pneumolysin mutant strains (ΔPLY). B) MeT-5A cells were treated with conditioned media derived from *S*. *pneumoniae* ΔPLY strains. Cell viability was determined 24 hr post-treatment * Denotes significantly higher than vehicle control.

We extended the dose response of MeT-5A cells to D39 ΔPLY and confirmed that D39 ΔPLY failed to induce cell death even at concentrations up to 5 x 10^7^ CFU/mL (Fig 5A). Lower doses of TIGR4, ST3 and ST23F ΔPLY strains still induced MeT-5A cell death albeit to varying degrees. TIGR4 ΔPLY induced MeT-5A cell death at all doses tested (Fig 5B). In contrast, no cytotoxic effect was observed for concentrations up to 10^5^ CFU/mL and 10^4^ CFU/mL for ST3 and ST23F ΔPLY strains, respectively (Fig 5C and 5D). Heat-killing of TIGR4, ST3 and ST23F ΔPLY strains abolished the cytotoxic activity of the bacteria on MeT-5A cells (Fig 6A). Further, treatment of MeT-5A cells with conditioned media from TIGR4, ST3 and ST23F ΔPLY strains significantly induced cell death (39.9%, 82.65% and 80.8%, respectively compared to vehicle controls; p < 0.01 for all; Fig 6B). Conditioned media from the TIGR4 ΔPLY strain induced cell death to a notably lesser extent than ST3 and ST23.

**Fig 7:**
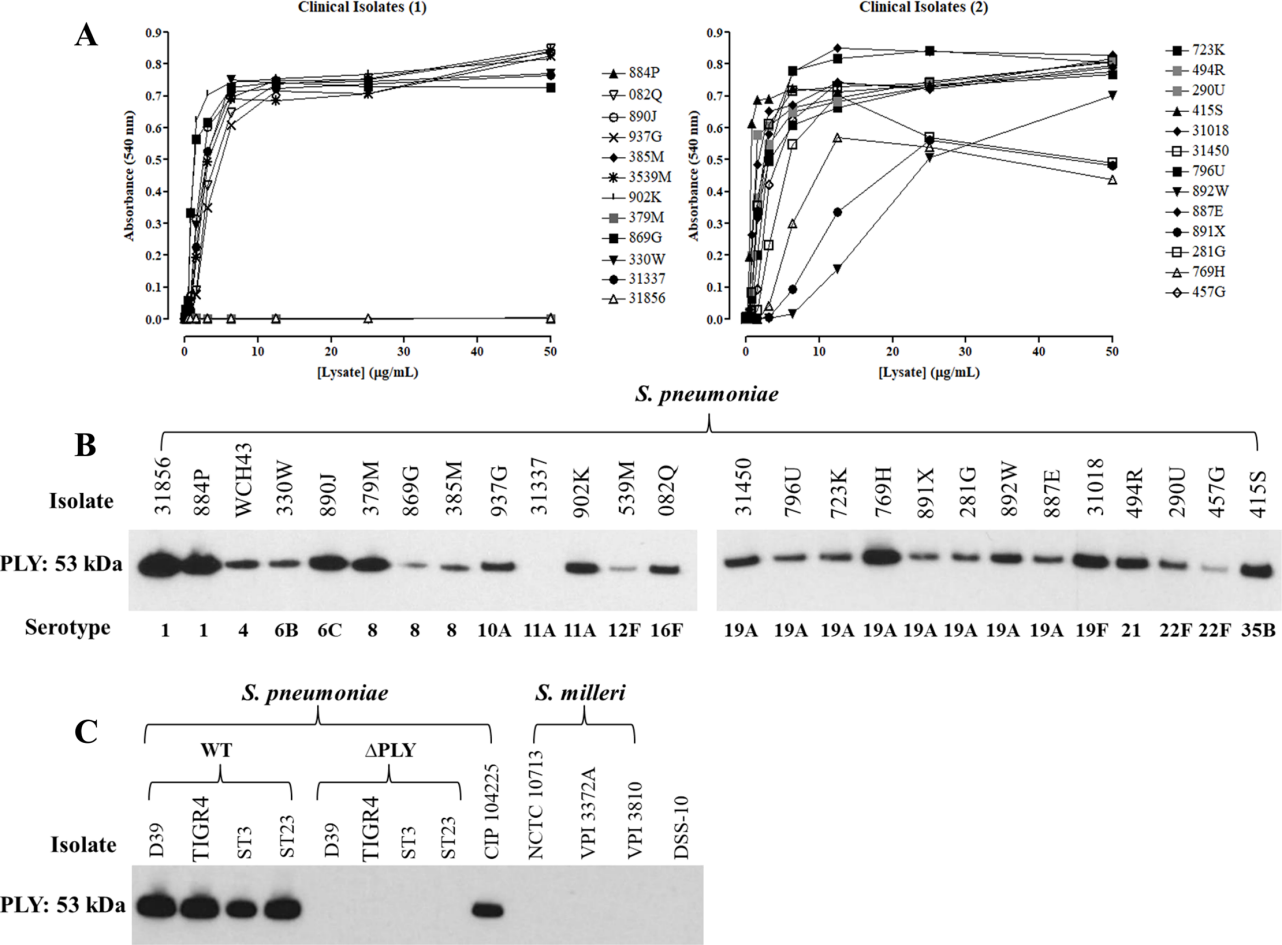
Hemolytic activity and pneumolysin expression in bacterial isolates. A) Lysates isolated from clinical *S*. *pneumoniae* isolates were assessed for cytolytic activity using an erythrocyte hemolysis assay. Pneumolysin protein expression was assessed by Western blot in lysates obtained from B) clinical and C) wild type and ΔPLY *S*. *pneumoniae* isolates strains and *S*. *milleri* and *S*. *sanguinis* strains.

To determine whether a secreted product was also responsible for the mesothelial cell cytotoxicity observed by clinical bacterial isolates, we quantified their hemolytic activity (Fig 7A). Most isolates produced cytolytic proteins, albeit to varying degrees. Combined with the results observed for the reference, wild type and ΔPLY strains, this added further support to a role for pneumolysin in mesothelial cell killing. We therefore assessed pneumolysin protein expression in the *S*. *pneumoniae* clinical bacterial isolates by Western blot (Fig 7B). All but one (31337) of the *S*. *pneumoniae* clinical isolates were positive for pneumolysin expression. Pneumolysin protein expression was also confirmed in the wild type but not the ΔPLY *S*. *pneumoniae* or *S*. *milleri* and *S*. *sanguinis* strains (Fig 7C).

## Discussion

Since first being described over 2000 years ago, pleural infection remains a major respiratory illness [20], and its global incidence is rising [1] despite advances in antibiotics and vaccination [21, 22]. Our understanding of the underlying pathobiology of this disease remains limited and although mesothelial cells are the most abundant cell type within the pleural cavity and *S*. *pneumoniae* is one of the most common causative agents of empyema, the host/pathogen interactions between these cells have seldom been studied.

Mesothelial cells can phagocytoze invading bacteria, release a large range of pro-inflammatory cytokines to recruit immune cells to the pleural cavity and induce fibrogenic reactions and septations/loculations to contain unwanted material within the pleural cavity. The ability of bacteria to compromise the mesothelial barrier (by killing mesothelial cells) will aid entry into, and subsequent bacterial proliferation within, the pleural cavity [23–25].

We showed, for the first time, that *S*. *pneumoniae* potently induced death of pleural mesothelial cells. These findings were validated using a large number of clinical isolates. This result was consistent across all reference strains tested and could be reproduced in 92% of the clinical isolates tested. Furthermore, mesothelial cell death occurred rapidly (<2 hrs) after exposure to *S*. *pneumoniae*, suggesting damage of the mesothelial barrier may be an early event in the onset of pleural infection. This potent cytotoxic effect on mesothelial cells was not observed following treatment with the other strains of common empyema bacteria we tested [8–10, 26].

Using several approaches, we confirmed that mesothelial cell killing was mediated by bacterial product(s) released by live *S pneumoniae*. The particular product(s) however appeared to vary among different strains of *S pneumoniae*. In the *S*. *pneumoniae* D39 strain, the cytotoxicity to mesothelial cells was mediated by pneumolysin and could be abolished by using *S*. *pneumoniae* mutant strains that lacked the pneumolysin gene (ΔPLY strains). Various common empyema pathogens, including *S*. *pneumoniae* strains, vary in their degree of pleural cytotoxicity but this study is the first to demonstrate this phenomenon towards mesothelial cells. Our data suggests that this may be due, in part, to the employment of separate pneumolysin-dependent and/or -independent mechanisms in order to mediate mesothelial cell death.

Pleural infection is traditionally viewed as an extension of lung parenchymal infection during pneumonia. In our published murine model of pneumococcal empyema, inhalation of *S*. *pneumoniae* induced histologic consolidation of the lung parenchyma and spread rapidly from the peri-bronchial areas to infect the visceral pleural mesothelial cells and enter into the pleural cavity. It is therefore probable that different microbes may employ alternate route(s) of entry to the pleural cavity. *S milleri*, for example, is a common causative agent for empyema, but rarely causes pneumonia. Computed tomography studies confirmed that a subgroup of empyema patients had no evidence of lung parenchymal infection. Our finding of high lethality of *S*. *pneumoniae* against mesothelial cells supports its direct invasion of the pleura through breaching the visceral mesothelium via translocation [14]. Our observation may also lend support to the hypothesis that other common microbes infect the pleura via different mechanisms. Non-cytotoxic bacteria, including *S*. *aureus* and viridans streptococci likely employ additional virulence strategies against mesothelial cells during pleura infection. These could include stimulation of cytokine release [24, 27–29], induction of mesothelial permeability [24] and cellular internalization and intracellular survival [14, 23, 30].

Pneumolysin is a cholesterol-dependent cytolysin, which forms large pores in membranes resulting in cell lysis and invasion of *S*. *pneumoniae* into host tissues [31]. We demonstrated that mesothelial cells are susceptible to pneumolysin-induced cell death when treated with pneumolysin. This was further confirmed when *S*. *pneumoniae* D39 ΔPLY failed to cause death of mesothelial cells. This result is consistent with other studies reporting attenuated cytotoxicity using pneumolysin mutated D39 [32, 33]. However, three other *S*. *pneumoniae* strains tested (TIGR4, ST3 and ST23F) were significantly less affected by pneumolysin gene mutation (ΔPLY). These ΔPLY strains produced a significantly weaker cytotoxic effect than the wild type strains, suggesting a role for pneumolysin-independent mechanism(s) in mesothelial cell death. Though the precise nature of these additional mechanisms was outside the scope of our study, this would suggest that both a capsular component/s and a secreted product (other than pneumolysin) may be required. Our results using heat-killed bacteria and conditioned media from the ΔPLY strains also favored the role of additional bacterial secreted product(s), rather than direct toxicity (e.g. via internalisation of *S*. *pneumoniae*) [34]. Given the absence of hemolytic activity in the ΔPLY strains, the secreted factor likely mediates cell death independent of direct cell lysis. Recently, *S*. *pneumoniae* ClpP protease was shown to induce apoptosis of human neuroblastoma cells [35]. Similarly, pneumococcal cell wall components released as a result of autolysis during growth are cytotoxic to some cell types [32, 36, 37]. These mechanisms warrant exploration.

There are limitations to our study. Although MeT-5A cells are considered the standard for mesothelial cell related studies, due to the considerable challenge of isolating primary cells in sufficient quantitites to establish reproducible *in vitro* models, the transformed nature of the cells may potentially confound interpretation of results. In addition, we investigated the direct cytotoxic effects of different bacteria on mesothelial cells *in vitro*, which did not incorporate any interactions of the bacteria that may initiate or augment cytotoxicity from other immune cells *in vivo*. Also, the precise impact of this lethality and any potential in therapeutic intervention need to be explored in animal models and human studies in future. In addition, intra-strain differences in *S*. *aureus*-induced cell death of peritoneal mesothelial cells [25] and a larger number of *S*. *aureus* and *S*. *milleri* strains need to be studied to make a firm conclusion of differences in their behavior from *S*. *pneumoniae*.

In conclusion, our results demonstrate that *S pneumoniae* is a potent inducer of pleural mesothelial cell death. Pneumolysin, although necessary for some *S*. *pneumoniae* strains, is not the sole pneumococcal mediator responsible for mesothelial cytotoxicity. Future studies are required to elaborate the relevance of these findings *in vivo*.
